# Effectiveness of game-based digital intervention for attention-deficit hyperactivity disorder in children and adolescents: a systematic review and meta-analysis using Beard and Wilson’s conceptualization of perception in experiential learning

**DOI:** 10.1007/s00787-025-02788-5

**Published:** 2025-06-16

**Authors:** Haesun Lee, Seungjin Lee, Mina Hwang, Kyungmi Woo

**Affiliations:** 1 Seoul National University College of Nursing, 103 Daehak-ro, Jongno-gu, Seoul, 03080 Republic of Korea; 2 Seoul National University Bundang Hospital, Seongnam, Republic of Korea; 3 The Research Institute of Nursing Science, Seoul National University, Seoul, Republic of Korea; 4 Center for Human-Caring Nurse Leaders for the Future by Brain Korea 21 (BK 21) Four Project, College of Nursing, Seoul National University, Seoul, Republic of Korea

**Keywords:** Adolescents, Attention-deficit hyperactivity disorder, Digital therapeutics, Game-based intervention, Meta-analysis, Systematic review

## Abstract

Attention-deficit hyperactivity disorder (ADHD) affects 8% of children worldwide, making it one of the most prevalent neurodevelopmental disorders. Traditional treatments, such as pharmacological and behavioral therapies, often have limitations. This study examined the effects of game-based digital interventions on specific cognitive and behavioral functions in children with ADHD, guided by experiential learning theory (ELT). We utilized ELT to synthesize results from various studies on game-based digital interventions across three dimensions: behavioral, cognitive, and affective responses. ELT also provided a framework for categorizing the gaming elements and strategies embedded in each intervention, facilitating the transformation of experience into learning. The systematic review included randomized controlled trials (RCTs) that assessed game-based digital health interventions targeting ADHD symptoms in children and adolescents. Studies focusing on non-digital or non-game-based interventions were excluded. A search was conducted across Embase, CINAHL, Cochrane Library, ProQuest, PsycINFO, and PubMed, with the final search date of October 20, 2024. The risk of bias in the included studies was assessed following PRISMA guidelines using the RoB 2 tool. Studies were analyzed using fixed-effects or random-effects models based on heterogeneity. The final analysis included eight RCTs, most of which focused on the cognitive effects of game-based interventions for ADHD. The meta-analysis revealed that game-based interventions significantly improved cognitive functions: (a) visuospatial short-term memory, as measured using the Corsi Block Tapping Task Forward (mean difference = 0.67; 95% confidence interval = [0.25, 1.10]), and (b) visuospatial working memory, as measured using the Corsi Block Tapping Task Backward (mean difference = 0.47; 95% confidence interval = [0.05, 0.89]). However, effects on behavioral aspects such as inhibition and monitoring, assessed using the BRIEF, were not statistically significant, suggesting limited behavioral improvement following the interventions. This study integrates the strategies and effects of game-based digital interventions for children and adolescents with ADHD within the ELT framework, highlighting the need to develop interventions that address not only cognitive deficits but also emotional and social challenges.

**Supplementary Information:**

The online version contains supplementary material available at 10.1007/s00787-025-02788-5.

## Introduction

A meta-analysis published in 2023, which included studies conducted between 2007 and 2022, reported that the global prevalence of ADHD in children and adolescents is 8% [1], making it one of the most prevalent neurodevelopmental disorders in this population. The incidence of ADHD, characterized by symptoms such as inattention, hyperactivity, and impulsivity, has been steadily increasing in recent years. Children with ADHD often experience comorbid mental health conditions, including depression, anxiety disorders, obsessive compulsive disorder [2], and, in some cases, alcohol and substance abuse [3]. Early treatment and management are critical, as ADHD can significantly impact academic performance, interpersonal relationships, and overall quality of life.

Treatment for ADHD typically includes pharmacological and behavioral therapies [4]. However, pharmacological treatments raise concerns over side effects, adherence issues, and resistance from patients or caregivers [5, 6]. Furthermore, medications alone often fail to address the full spectrum of ADHD symptoms [7]. In recent years, non-pharmacological interventions, such as behavioral therapy, social skills training, and parental training, have gained attention [4], though they require consistent and accessible implementation owing to their short-lasting effects [8]. As a result, digital technology–based interventions have emerged as promising alternatives, offering sustained accessibility and innovative therapeutic approaches.

Among these, game-based interventions are increasingly recognized as effective digital strategies for capturing children’s attention and promoting learning and behavioral changes [9]. Notably, the US Food and Drug Administration recently approved EndeavorRx^®^, a prescription-only game-based digital therapeutic, highlighting the clinical potential of such interventions [10]. Games present novel opportunities to enhance attention span and self-regulation skills in children with ADHD, using engaging elements to encourage voluntary participation [11–13]. Through repetitive learning and immediate feedback, game-based interventions have been shown to boost motivation for learning [14]. In addition to providing extra learning opportunities, games create a safe environment for learning from mistakes, an essential component of experiential learning [15].

The experiential learning theory (ELT) suggests that learning is a process in which knowledge is created by transforming experiences [16]. This theory emphasizes that knowledge is acquired through passive absorption, active engagement, and reflection on experiences. Game-based digital interventions for children with ADHD align well with ELT by offering immersive, interactive environments that encourage active participation, reflection, and problem-solving. These interventions facilitate the assimilation and transformation of experiences, which are key components of experiential learning [17]. Current game-based interventions such as EndeavorRx (Akili Interactive), ATENTIVmynd (ATEN TIV), SparkRx (Limbix), and Canvas Dx (Cognoa), use various strategies to improve ADHD symptoms, focusing on the interactive relationship between learning processes and cognitive, behavioral, and emotional changes as outlined in ELT [18].

While several systematic reviews and meta-analyses have assessed the effects of non-pharmacological interventions on ADHD [19, 20], to our knowledge, few studies have specifically focused on game-based interventions for children and adolescents with ADHD or performed meta-analyses to quantify treatment effectiveness [14, 21]. Moreover, comprehensive evaluations of the impact of different game types and strategies used in these interventions on ADHD symptoms remain limited. Therefore, it is essential to explore how game-based interventions influence ADHD symptoms and investigate the cognitive and behavioral strategies employed in such interventions.

Given the limitations of previous research, this study aims to examine the cognitive and behavioral effects of game-based digital therapies on ADHD treatment, with a focus on how these interventions affect attention, impulse control, and behavioral regulation.

## Methods

Before conducting the review, we searched the International Prospective Register of Systematic Reviews (PROSPERO) and the Cochrane Library to confirm that no prior or ongoing systematic reviews existed on game-based digital therapeutics for ADHD in children and adolescents. Based on prior literature, our research team defined game-based digital interventions as digital health tools, computer-assisted therapies, and mobile applications that incorporate games. This study adhered to the Preferred Reporting Items for Systematic Reviews and Meta-Analyses (PRISMA) statement [22], a widely used framework that ensures transparent and thorough reporting of systematic reviews. The study protocol was registered and published by PROSPERO (registration number: CRD42024629174).

### Information sources

We searched electronic databases, including Embase, PubMed, the Cochrane Library, CINAHL, PsycINFO, and ProQuest, for studies published before October 2024. No limits were applied to the database search to capture a broad range of relevant articles. In addition, a citation-tracking search was performed to identify additional pertinent articles.

### Search strategy

To ensure a systematic and thorough literature search, we developed a search strategy based on our PICO question for October 2024. The strategy included three key concepts: (1) ADHD, (2) children and adolescents, and (3) game-based digital interventions. Game-based digital interventions were searched within three categories: digital health, computer-assisted therapy, and mobile applications combined with games. The search terms were identified from previous studies and then reviewed and refined by a medical librarian to ensure their accuracy and completeness. Three reviewers (HL, MH, and SL) conducted the searches. We used keywords and combinations, including indexed terms such as MeSH terms, CINAHL headings, and text words, to search the electronic databases. The literature search was conducted from October 1 to October 20, 2024. Supplementary File 1 contains the full search strategy and provides a summary of the PubMed search strategy and its results.

### Eligibility and inclusion criteria

Studies were included if they (a) were primary scholarly articles focused on game-based digital health interventions for treating ADHD symptoms, (b) were randomized controlled trials (RCTs), (c) involved children (under 18 years) or adolescents (10–19 years) diagnosed with ADHD, and (d) were published in English. Studies were excluded if they (a) were theoretical, discussion, review, or meta-analysis articles; (b) included children or adolescents with psychiatric conditions other than ADHD; (c) focused on non-digital health interventions, non-game-based digital interventions (e.g., web-based educational programs or non-gamified applications), or treatments unrelated to ADHD symptom management (e.g., interventions for speech and language disorders); or (d) focused on assessment or diagnostic methods rather than treatment.

### Study selection

**Fig. 1:**
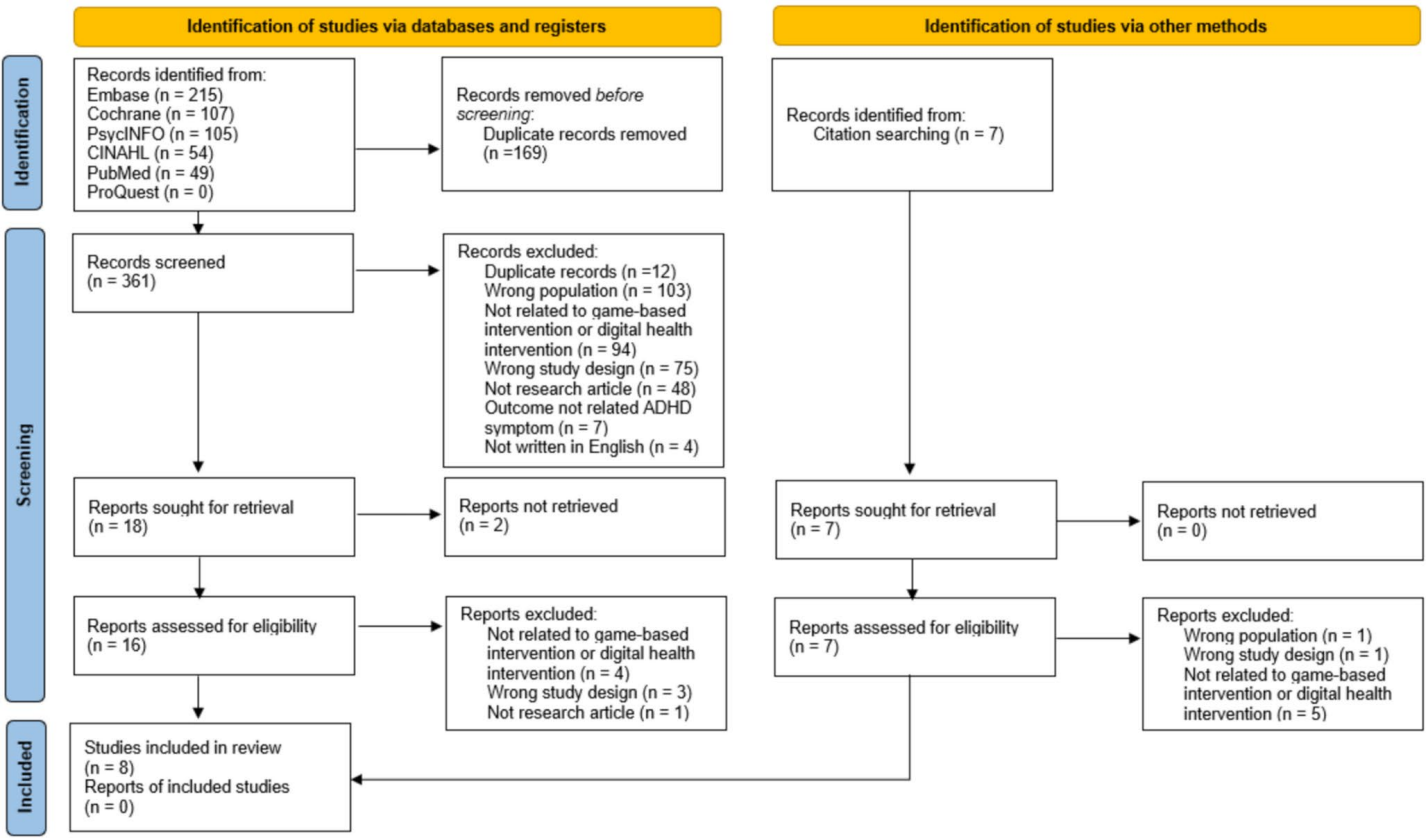
PRISMA flow chart for literature selection

Figure 1 presents the PRISMA flow diagram, which outlines the study selection process: (1) identifying the total number of studies from all electronic databases through manual searching, (2) evaluating the studies for eligibility, (3) selecting studies for inclusion in the review, and (4) excluding studies based on the exclusion criteria. We conducted a systematic search using the Rayyan web-based tool [23] to streamline the systematic review process. All screening steps were independently performed by three reviewers and cross-checked in pairs (HL-MH, MH-SL, and SL-HL) based on the eligibility criteria. Disagreements between reviewers were resolved through discussions until consensus was reached.

### Data extraction

Eight full-text articles were distributed evenly among three reviewers (HL-MH, MH-SL, and SL-HL), and data were independently extracted from each article. The extracted data were then cross-checked in pairs (HL-MH, MH-SL, and SL-HL). The research team created an abstract format using Microsoft Excel spreadsheets and a coding manual to maintain coding consistency. The data extraction form was carefully designed to align with the research questions. The following study characteristics were included: author, year, purpose, study design, participant information, intervention, measurement, and main outcomes. We aimed to develop a comprehensive abstraction format by referencing previous studies. Any disagreements were resolved by discussion among the reviewers until consensus was reached.

### Quality appraisal

We assessed the methodological quality of the eight selected studies using the revised Cochrane Risk-of-Bias Tool for Randomized Trials (RoB 2) [24]. RoB 2 was specifically designed to evaluate bias in RCTs across five domains: (1) bias arising from the randomization process, (2) bias due to deviations from intended interventions, (3) bias due to missing outcome data, (4) bias in the measurement of the outcome, and (5) bias in the selection of the reported results. This comprehensive evaluation makes RoB 2 a suitable tool for assessing the internal validity of the included studies. The eight studies were assigned to three pairs of reviewers (HL-MH, MH-SL, and SL-HL), with each pair independently evaluating their assigned studies. Any discrepancies were resolved through discussions until consensus was reached. Each study was then given a final risk rating: low risk, some concern, or high risk.

### Quantitative synthesis

Meta-analysis was conducted using RevMan (version 7.2) [25]. Only outcomes measured with the same instruments in at least two studies were included in the analysis. The meta-analysis included eight subscales of the Behavior Rating Inventory of Executive Function (BRIEF) assessed via parent reports, as well as working memory as measured using the Corsi Block Tapping Task (CBTT). Effect sizes were calculated as mean differences (MDs) with 95% confidence intervals (CIs). Statistical heterogeneity was assessed using the I² statistic, with values greater than 50% indicating substantial heterogeneity [26]. Given the diversity of interventions and ADHD subtypes, a random-effects model was used for the meta-analysis [27]. However, when only two studies were available for analysis, a fixed-effects model was applied to estimate heterogeneity and address the limitations of the random-effects model in such cases [28].

### Conceptual frameworks

**Fig. 2:**
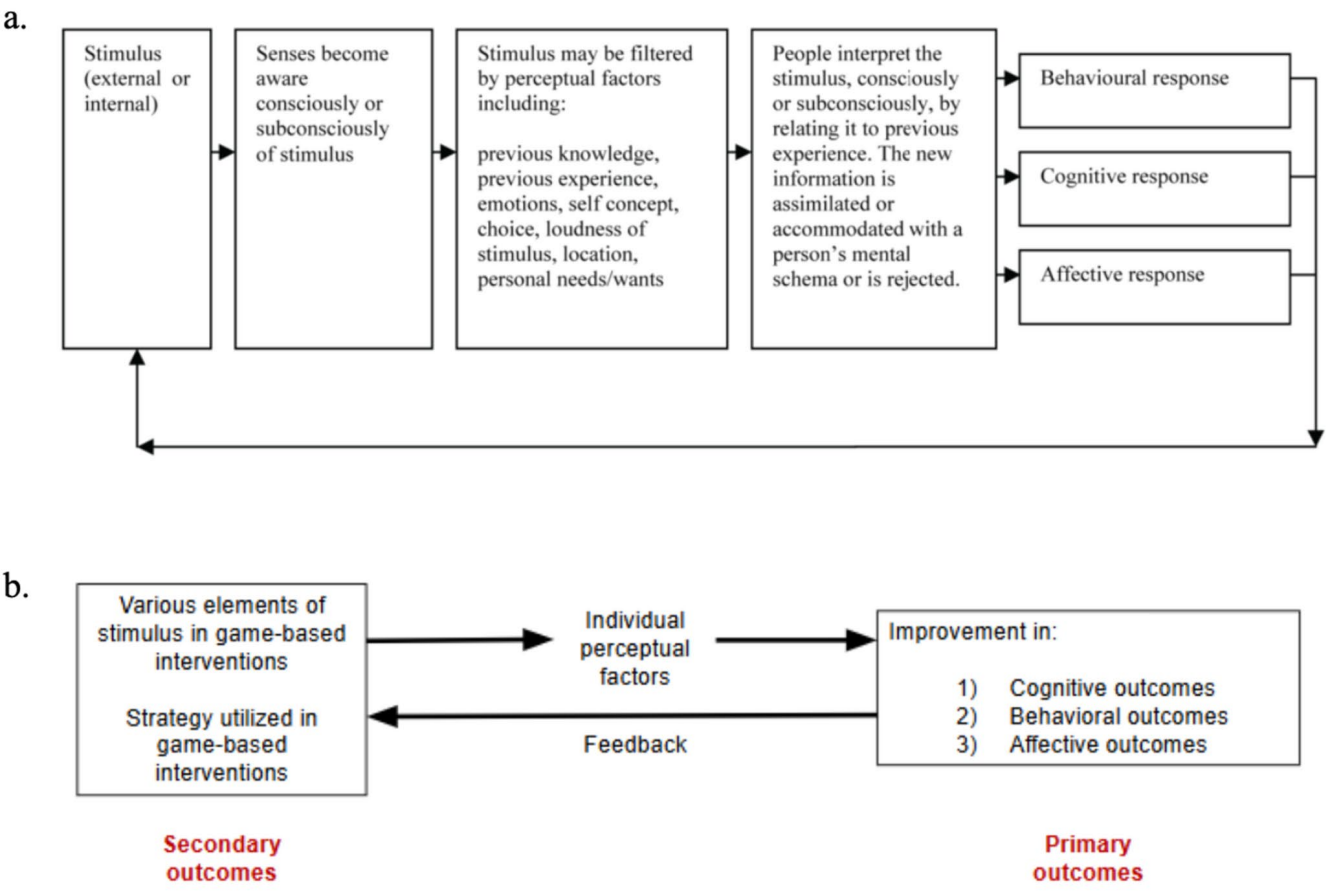
Conceptual frameworks. Based on Beard and Wilson’s conceptualization of perception in experiential learning theory (**a**), this study organized the conceptual framework (**b**) including primary outcomes and secondary outcomes

This study organizes the contents and effects of game-based interventions through Beard and Wilson’s ELT. This model offers a robust framework to understand how learning occurs through active engagement and reflection on personal experiences (Fig. 2). Specifically, ELT is useful for examining how game-based interventions foster experiential learning through dynamic interaction, reflection, and the application of new skills [17]. ELT’s cyclical process—comprising concrete experience, reflective observation, abstract conceptualization, and active experimentation—aligns well with the interactive nature of game-based learning, where participants learn by doing, reflecting on their actions, and adjusting their behaviors. The theory conceptualizes the impact of interventions across three dimensions: (1) behavioral, (2) cognitive, and (3) affective responses. This makes ELT an ideal theoretical lens for systematically reviewing game-based programs for ADHD, as it offers a deeper understanding of how these interventions support cognitive, emotional, and behavioral development, while providing a structured framework for synthesizing diverse findings across studies.

## Results

### Selected articles

A total of 530 studies were identified. After excluding 169 duplicates, the research team reviewed the titles and abstracts of 361 non-duplicate articles. Of these, 343 studies were excluded because they did not meet the criteria (e.g., incorrect population or unrelated to game-based interventions). The remaining 18 articles underwent full-text review, with 2 being study protocols. After excluding these 2 studies, 16 articles, along with 7 additional studies identified through citation tracking, remained for full-text screening. From these 23 articles, 15 were excluded owing to issues with publication type, study design, or intervention type not meeting the inclusion criteria. Ultimately, eight studies were included in this review.

### Risk of bias of individuals studies

**Fig. 3:**
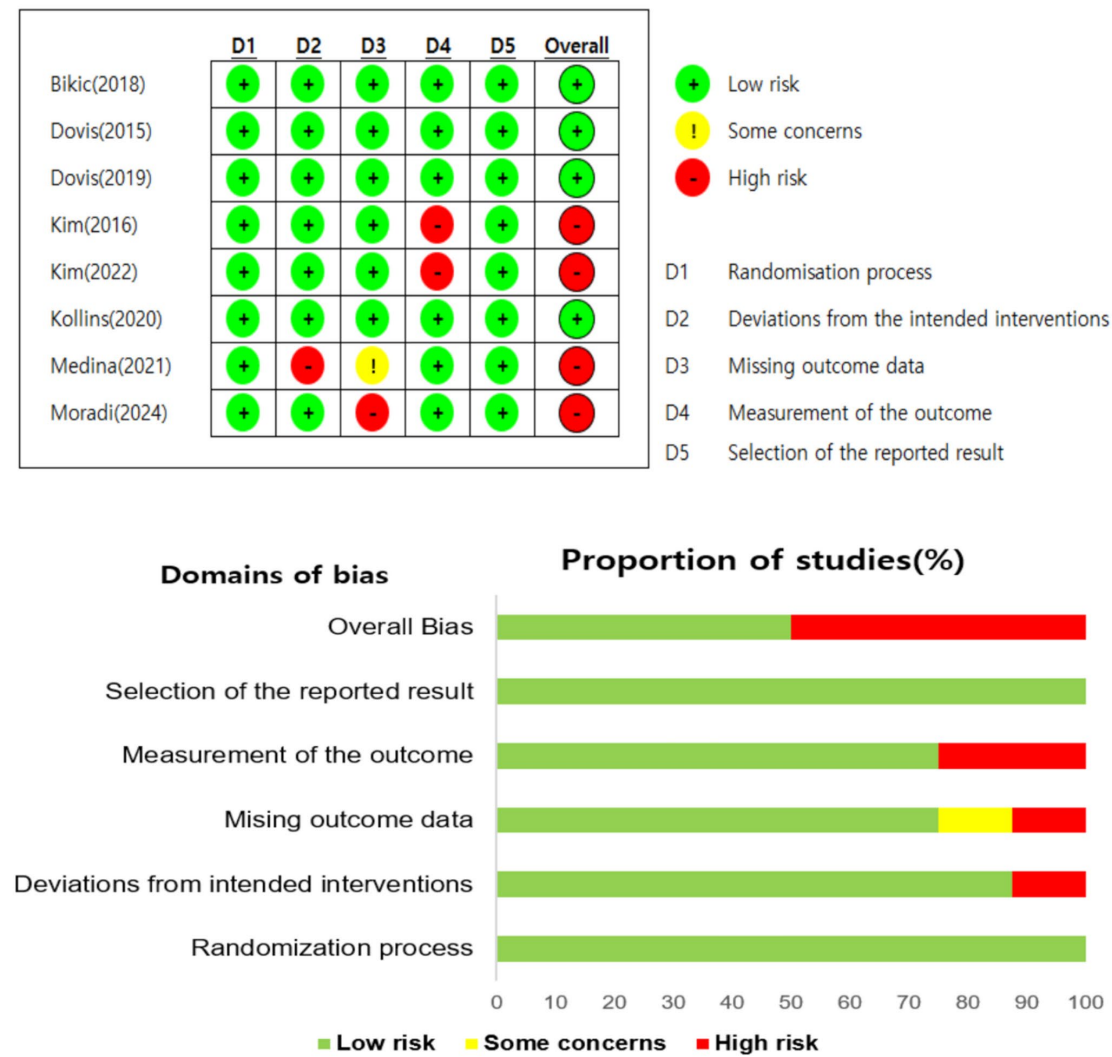
Quality appraisal and summary of included studies

Figure 3 illustrates the risk of bias for each study. The quality of the studies was assessed as low or high (*n* = 4 and *n* = 4, respectively). Low-quality ratings were primarily owing to issues with outcome measurement and the handling of missing data. All studies used a randomization process. Regarding deviations from intended interventions, one study exhibited a high risk of bias owing to unclear reasons for a high dropout rate [29]. In terms of handling missing data, one study had a high risk of bias, as the missing data seemed dependent on the true value of the outcome [30]. Another study showed some concern regarding missing data, but the proportion and reasons for missing data were balanced across groups [29]. For outcome measurement bias, two studies were rated high owing to the absence of validated measurements [31, 32].

### Systematic review of game-based intervention effects

#### General characteristics of reviewed papers

All eight studies were RCTs. Seven employed a two-arm trial design [29–35], while one used a three-arm design [36]. These studies were conducted between 2015 and 2024 in seven countries: Belgium, Denmark, Iran, the Netherlands, South Korea, Spain, and the United States. A total of 801 school-aged children and adolescents diagnosed with ADHD participated, with sample sizes ranging from 29 to 348. The age range of the participants in the studies was between 6 and 13 years, and the number of male participants was approximately 4 to 6 times greater than that of female participants in each study. Most studies were conducted in the participants’ homes [29, 31–36], with only one conducted in a school setting [30]. The detailed characteristics of the reviewed studies are summarized in Table 1.

**Table 1:** General characteristics of reviewed studies

No	Author (year)	Country	Aims	Study design	Setting	Participant characteristics				
						Sample Size (M: F)	Age range	Type of ADHD^a^	Inclusion IQ^e^	Medication usage
1	Bikic (2018)	Denmark	To identify computer games targeting a number of cognitive functions might have a better effect on the symptoms and cognition than interventions targeting single or a few cognitive functions(working memory)	RCT^f^	Community (home-based)	EG^g^: 35 (29:6) CG^h^: 35 (30:5)	6 ~ 13	ADHD-H^b^ (*n* = 4) ADHD-I^c^I (*n* = 30) ADHD-C^d^(*n* = 35)	≥ 80	Yes
2	Dovis (2015)	Netherland	To investigate the efficacy of a gamified, 5-week, home-based, multiple EF^i^ training intervention titled Braingame Brian in children with ADHD-Combined type	RCT	Community (home-based)	EG: 31 (25:6) CG: 30 (24:6)	8 ~ 12	ADHD-C	≥ 80	Yes
3	Dovis (2019)	Netherland	To determine whether pre-training EF capacity is a moderator of near(EF performance) and far transfer effects(ADHD symptoms and parent-rated EF behavior) of a gamified, 5-week, home-based, EF training intervention titled Braingame Brian	RCT	Community (home-based)	EG: 31 (25:6) CG: 30 (24:6)	8 ~ 12	ADHD-C	≥ 80	Yes
4	Kim (2016)	Netherland, Belgium	To identify a) improvement on primary outcome measures of time management, planning/organizing, and cooperation skills compared to participants in the crossover control group b) improvement on working memory, social skills (e.g., responsibility, assertiveness, and self-control and self-efficacy	RCT	Community (home-based)	EG: 88 (70:18) CG: 82 (67:15)	8 ~ 12	ADHD-H (*n* = 6) ADHD-I (*n* = 38) ADHD-C (*n* = 126)	≥ 80	Yes
5	Kim (2022)	Korea	To evaluate the effectiveness of a game-type NeuroWorld cognitive training program for children with ADHD using digital therapy	RCT	Community (home-based)	EG: 15 (12:3) CG: 15 (11:4)	6 ~ 13	NA	NA	Yes
6	Kollins (2020)	USA	To assess whether AKL-T01 improved attentional performance in pediatric patients with ADHD.	RCT	Community (home-based)	EG: 180 (NA) CG: 168 (NA)	8 ~ 12	NA	≥ 80	No
7	Medina (2021)	Spain	To identify whether a case-based reasoning digital training regimen would be effective in an ADHD child population	RCT	Community (home-based)	EG: 15 (13:2) CG: 14 (12:2)	8 ~ 11	ADHD-C	NA	Yes
8	Moradi (2024)	Iran	To answer whether neurofeedback in combination with cognitive games would improve the perception, attention, the working memory of children with ADHD	RCT	Community (school)	EG: 16 (16:0) CG: 16 (16:0)	NA	ADHD-H (*n* = 8) ADHD-I (*n* = 5) ADHD-C (*n* = 19)	≥ 85	Yes

^a^ADHD attention-deficit/hyperactivity disorder; ^b^ADHD-H attention-deficit/hyperactivity disorder-hyperactive; ^c^ADHD-I attention-deficit/hyperactivity disorder-inattentive; ^d^ADHD-C attention-deficit/hyperactivity disorder-combined; ^e^IQ Intelligence Quotient; ^f^RCT Randomized Controlled Trial; ^g^EG Experimental Group; ^h^CG Control Group; ^i^ EF executive functioning

#### Objectives of included studies

Among the studies included in this review, the majority primarily aimed to examine the cognitive effects of game-based interventions for ADHD. Specifically, 62.5% of the studies aimed to improve cognitive processes [30, 32, 34–36], 12.5% focused on enhancing daily life functioning [31], and 25% adopted a multi-domain approach [29, 33], targeting both cognitive processes and behavioral symptoms. The most commonly studied cognitive subdomains were response inhibition (*n* = 5) and working memory (*n* = 5), followed by cognitive flexibility (*n* = 3), sustained attention (*n* = 2), and cognitive control (*n* = 2). Additional subdomains included information processing speed, multiple simultaneous attention, category formation, and pattern recognition. While none of the studies specifically targeted the psychological effects of game-based interventions for ADHD, two studies assessed socio-psychological outcomes such as quality of life and self-efficacy.

#### Methodological characteristics and interventions

None of the studies specified a randomization method. Four studies implemented double blinding for the experimental and control conditions [30, 34–36], two used single blinding [29, 33], and the remaining two did not use any form of blinding [31, 32]. Most studies (75%) delivered interventions via computer [29–31, 33, 34, 36], while two studies (25%) used tablets as the platform [32, 35]. All game-based interventions utilized serious games with mission-guided tasks aimed at improving cognitive function. Participants were required to maintain attention, select targets, retain task-relevant information, and inhibit responses to stimuli. The number of intervention sessions ranged from 20 to 100, conducted over a period of 4 weeks to 3 months. Five studies reported session durations ranging from 15 to 45 min. Over half (62.5%) evaluated participants only at the end of the interventions [29, 30, 32, 33, 35], while 37.5% included follow-up assessments [31, 34, 36]. Among the follow-up studies, two assessed participants at 3 months to evaluate long-term effects, and one study conducted follow-up at 10 weeks. Table 2 summarizes the components of the interventions in each study in detail.

**Table 2:** Interventions, measurements and key outcomes in reviewed studies

No	Author (year)	Game name (Tool)	Targeting domain	Intervention Group	Controlled Group	Duration of intervention	Measured outcomes	Main outcomes
1	Bikic (2018)	ACTIVATE™ (Computer)	Cognitive domain: a) Sustained attention b) Response inhibition c) Cognitive flexibility and control d) Speed of information processing e) Multiple simultaneous attentions f) Working memory g) Category formation h) Pattern recognition Behavioral symptoms	Treatment as usual + cognitive computer games part of ACTIVATE™ a) Catch the Ball b) Butterflies c) What Comes Next	Treatment as usual	40 min/session, 6 times/week, for 8 weeks	***Cognitive outcomes***: Cambridge Neuropsychological Test Automated Battery (CANTAB) 1. Visual, movement and comprehension difficulties: The Motor Screening Task (MOT) screening 2. Attentions tests: Attention switching task(AST), Rapid Visual Information Processing(RVP)† 3. Executive functions: Spatial working memory (SWM), Stockings of Cambridge (SOC), Intra-extra dimensional set shift (IED), Stop signal task(SST), Reaction time(RTI). ***Behavioral outcomes***: ADHD-Rating Scale-IV‡ Behavior rating inventory of executive function (BRIEF, full domain)‡ Weiss functional impairment rating scale-parent report form (WFIRS-P)	Significantly improve in: Accuracy in planning ↑ (also maintained over 12 months) No significant in: Cognitive outcomes except accuracy in planning Behavioral outcomes
2	Dovis (2015)	Brain game Brian (Computer)	Cognitive domain: Executive functioning a) Working memory b) Inhibition c) Cognitive -flexibility	Working Memory: a) Visuospatial short-term memory b) Updating and manipulation of information Inhibition task: designed to decrease the time needed to inhibit a prepotent response Cognitive-flexibility: designed to decrease the time a child needs to adapt his/her behavior when task-rules change	Placebo condition	35 ~ 50 min/session, 25 sessions, for 5 weeks	***Cognitive outcomes***: Stop Signal Reaction Time (SSRT) Stroop Test Corsi Block Tapping Task-forward, Corsi Block Tapping Task-backward Digit Span, Trail Making Test (TMT) Raven Colored Progressive Matrices ***Behavioral outcomes***: Disruptive Behavior Disorder Rating Scale (DBDRS) BRIEF (full domain) Sensitivity to Punishment and Sensitivity to Reward Questionnaire for children (SPSRQ-C) ***Others***: Pediatric Quality of Life Inventory The Home Situations Questionnaire	Significantly improve in: Working Memory ↑ Inhibition ↑ Visuospatial STM ↑ Pediatric Quality of Life ↑
3	Dovis (2019)	Brain game Brian (Computer)	Cognitive domain: Executive functioning a) Working memory b) Inhibition c) Cognitive flexibility	Working Memory: a) Visuospatial short-term memory b) Updating and manipulation of information Inhibition task: designed to decrease the time needed to inhibit a prepotent response Cognitive-flexibility: designed to decrease the time a child needs to adapt his/her behavior when task-rules change	Placebo condition	35 ~ 50 min/session, 25 sessions, for 5 weeks	***Cognitive outcomes***: Corsi Block Tapping Task-forward, Corsi Block Tapping Task-backward Stop Signal Reaction Time (SSRT) Trail making test (TMT) ***Behavioral outcomes***: DBDRS BRIEF(Working memory, Inhibit, and Shift)	Significantly moderate of: a) inhibition performance and cognitive flexibility performance on - Near transfer ↑ - Working memory ↑ - Performance ↑ b) working memory performance and cognitive flexibility performance - Far transfer ↑
4	Kim (2016)	Plan-It Commander (Computer)	Daily life functioning: a) Time management b) Planning/ organizing c) Cooperation skills	Internet-based serious game which consist of 2 components: a) Mission-guided game related to the learning goals of time management, planning/organizing, and cooperation skills b) Closed social community	Treatment as usual	Up to 65 min/session, 3 times/week, for 10 weeks	***Behavioral outcomes***: BRIEF(Plan/organize)† BRIEF(Working memory)‡ ***Social skills***: Social Skills Rating System(cooperation†, assertive‡, responsibility‡, self-control‡) ***Others***: Time management questionnaire† It’s About Time Questionnaire(IATQ)‡ Self-efficacy‡ Satisfaction‡	Significantly improve in: Time management skill ↑ Working memory ↑ Responsibility skills ↑ Not significant in: Planning/organization skill No difference in: Cooperation skill Social skills Self-efficacy
5	Kim (2022)	NeuroWorld DTx (Tablet)	Cognitive domain: a) Attention b) Working memory	Components of attention: a) Breathtaking glass bridge b) Find a friend in glass bridge c) Space travel in vortex d) Space travel Components of Working memory: a) Animals out of the yard b) Escape from the yard	Treatment as usual	Up to 30 min/day, 20 times, for 4 weeks	***Cognitive outcomes***: Comprehension attention test (CAT) ***Behavioral outcomes***: Korean Child Behavior Checklist (K-CBCL 6–18) ***Clinical ADHD symptoms***: Korean ADHD Rating Scale (K-ARS) Clinical Global Impression (CGI)	Significantly improve in: ADHD behavior symptoms Not significant in: Attention ADHD severity
6	Kollins (2020)	AKL-T01 (Tablet)	Cognitive domain: a) Attention b) Cognitive control processes	Video game-like interface displaying two tasks: a) Parallel (multitasking): a perceptual discrimination targeting task in which participants respond to the instructed stimulus targets and ignore the stimulus distractors b) Sensory motor navigation task in which participants continuously adjust their location to interact with or avoid positional target	Treatment as usual	25 min/session, 5 sessions/day, 5 days/week, for 4 weeks	***Cognitive outcomes***: Test of Variables of Attention Attention Performance Index (TOVA-API)† ***Behavioral outcomes***: BRIEF(Working memory, Inhibit)‡ ***Clinical ADHD symptoms***: Impairment Rating Scale (IRS)‡, ADHD-RS-IV‡, Clinical Global Impressions-Improvement (CGI-I)‡	Significantly improve in: Attention performance ↑ No difference in: ADHD symptoms Functional impairment
7	Medina (2021)	KAD_SCL_01 (Computer)	Cognitive domain: a) Inhibition Behavioral symptoms	AI-driven, game-based intervention: a) Composed of 14 types of games b) Game level is adapted based on a case-based reasoning algorithm	Placebo condition	15–20 min/session, 3 sessions/week, for 12 weeks	***Cognitive outcomes***: Commission score from Conners CPT (CPT-III)† Inhibition Test‡ Cognitive Flexibility Test‡ Corsi Block Tapping Test Wechsler Non-Verbal Scales(WNV)‡ Auditory Attention Test‡ Digit Span Test‡ Verbal Fluency Test‡ Symbol Search Test‡ Digit Symbol Substitution Test‡ ***Behavioral outcomes***: BRIEF‡, ***Clinical ADHD symptoms***: Evaluation of Attention Deficit and Hyperactivity Disorder (EDAH)‡	Significantly improve in: Inhibitory control ↑ Visuospatial working memory ↑
8	Moradi (2024)	SmartMind games (Computer)	Cognitive domain: a) Working memory b) Inhibitory control	Designed to reduce the severity of ADHD symptoms and enhance the frontal lobes function. a) The first and second parts do not require using hands. b) In the third part, the brain waves were examined in the games, which demand using the mouse. c) The fourth part involved DVD or CD.	Treatment as usual	45 min/session, 3 times/week, for 3 months	***Cognitive outcomes***: Time Perception Test Continuous Performance Test (CPT) Wechsler working memory test (WISC)	Significantly improve in: Short time perception ↑ Long time perception ↑ Attention ↑ Working memory ↑

†: Primary outcome in reviewed study, ‡: Secondary study on reviewed study

#### Gaming elements and strategies

Supplementary File 1 outlines the gaming elements and strategies used in the reviewed studies. Five studies (62.5%) incorporated rewards, such as points or badges, to motivate participation [31, 33–36]. Two distinct intervention approaches were identified regarding difficulty level: (a) two studies tailored the workload to individual performance [29, 35] and (b) three studies progressively increased the difficulty to test participants’ adaptive abilities under changing conditions [33, 34, 36]. Medina [29] used an artificial intelligence–driven algorithm to adjust difficulty levels. Three studies provided immersive environments within the games that allowed exploration and meaningful interactions [31, 34, 36]. In two studies by Dovis [34, 36], the main character “Brian” interacted with other game characters, while Kim [31] enabled players to help or communicate with one another via in-game messaging. Using these strategies, Kim [31] uniquely assessed the cooperation subscale as a component of social skills.

### Main outcomes

Cognitive functions, including executive function, working memory, inhibition, visuospatial short-term memory, attention, short-term memory, and long-term memory, improved significantly after the intervention. Regarding ADHD behavioral outcomes, findings were mixed: Kim [32] found a significant improvement in behavioral symptoms, while Bikic [33] observed no significant changes. Game-based interventions significantly improved time management and responsibility but did not impact social skills such as cooperation and assertiveness. No studies explored the affective effects of game-based interventions for ADHD.

#### Meta-analysis results of the game-based intervention effects

**Fig. 4:**
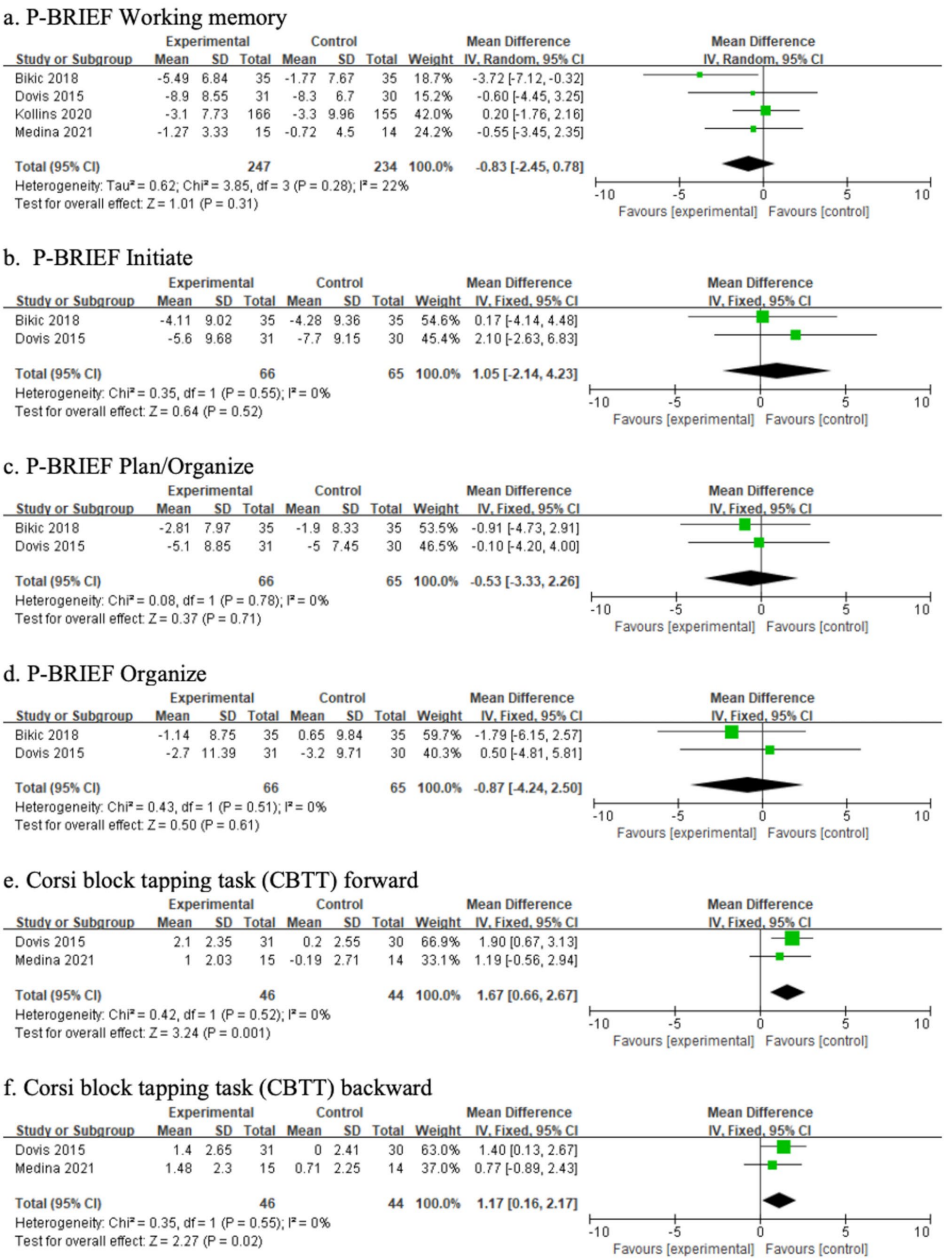
Cognitive results of meta-analysis

**Fig. 5:**
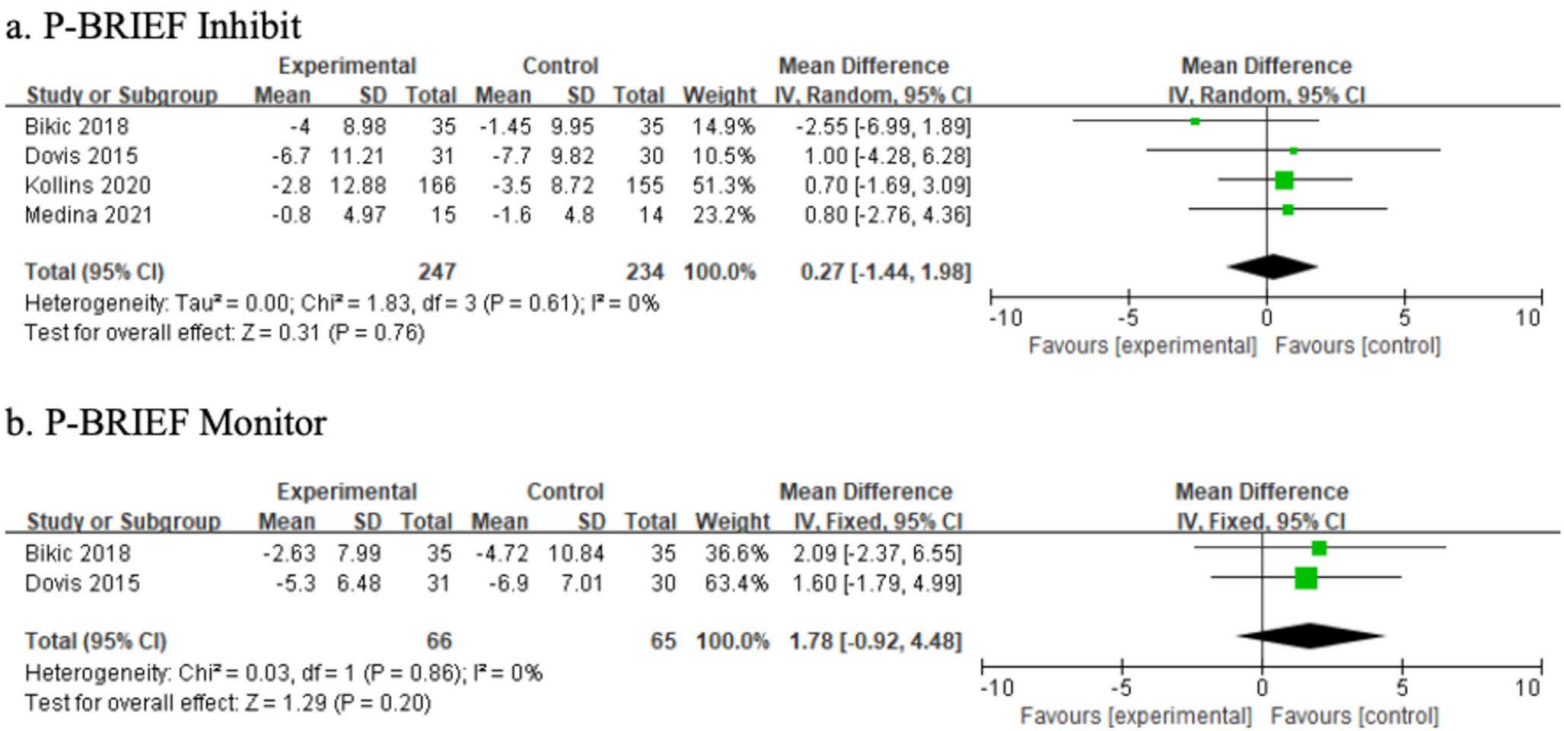
Behavior results of meta-analysis

**Fig. 6:**
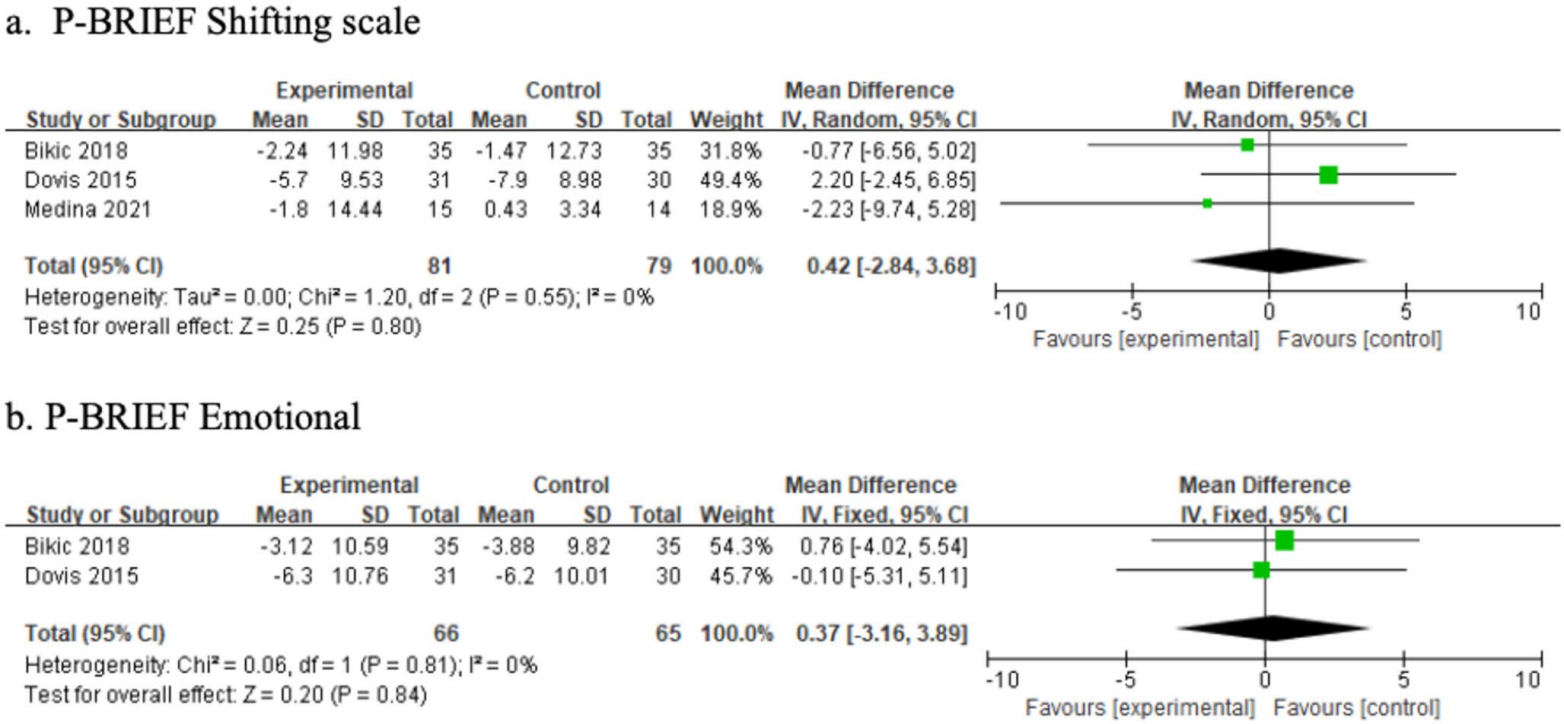
Affective results of meta-analysis

Gioia defined executive function as a collection of interconnected yet distinct abilities that regulate cognitive, behavioral, and emotional processes [37]. Based on Gioia’s confirmatory factor analysis of the BRIEF, the following latent factors were identified: (a) metacognition, encompassing initiation, working memory, planning/organization, material organization, and task monitoring; (b) emotional regulation, including shifting and emotional control; and (c) behavioral regulation, which involves self-monitoring and inhibition [37]. The measurements were grouped into cognitive, behavioral, and affective domains (Table 3). A meta-analysis integrating findings from prior studies, using the ELT framework, further examined the effects of game-based interventions across these domains. Figures 4, 5 and 6 present the results of the subgroup meta-analysis.

**Table 3:** Primary outcomes included in meta-analysis

Domain in Beard and Wilson’s ELT	Measurement	Number of studies	Studies
Cognitive responses	P-BRIEF Working memory	4	Bikic (2018), Dovis (2015), Medina (2021), Kollins (2020)
	P-BRIEF Initiate	2	Bikic (2018), Dovis (2015)
	P-BRIEF Plan/Organize	2	Bikic (2018), Dovis (2015)
	P-BRIEF Organization of materials	2	Bikic (2018), Dovis (2015)
	CBTT	2	Dovis (2015), Medina (2021)
Behavioral responses	P-BRIEF Inhibit	4	Bikic (2018), Dovis (2015), Medina (2021), Kollins (2020)
	P-BRIEF Monitor	2	Bikic (2018), Dovis (2015)
Affective responses	P-BRIEF Shifting scale	3	Bikic (2018), Dovis (2015), Medina (2021)
	P-BRIEF Emotional control	2	Bikic (2018), Dovis (2015)

#### Effects on cognitive aspect

The meta-analysis of game-based interventions on cognitive aspects included six subdomains: working memory, initiation, planning/organization, and material organization, measured using the BRIEF, as well as the CBTT Forward and Backward. Compared with the control group, game-based interventions were not effective in improving working memory (MD = − 0.83; 95% CI = [− 2.45, 0.78]), initiation (MD = 1.05; 95% CI = [− 2.14, 4.23]), planning/organization (MD = − 0.53; 95% CI = [− 3.33, 2.26]), or organizing materials (MD = − 0.87; 95% CI = [− 4.24, 2.50]). However, CBTT Forward (MD = 1.67; 95% CI = [0.66, 2.67]) and CBTT Backward (MD = 1.17; 95% CI = [0.16, 2.17]) showed significant improvements after the intervention, indicating that the interventions were effective in enhancing visuospatial short-term memory and visuospatial working memory. Figure 4 presents the results of the meta-analysis on cognitive outcomes.

#### Effects on behavioral aspects

The meta-analysis of game-based interventions on behavioral aspects included two subdomains: inhibition and monitoring, measured using the BRIEF. Compared with the control group, game-based interventions were not effective in improving inhibition (MD = 0.27; 95% CI = [− 1.44, 1.98]) or monitoring (MD = 1.78; 95% CI = [− 0.92, 4.48]). Figure 5 presents the results of the meta-analysis on behavioral outcomes.

#### Effects on affective aspect

The meta-analysis of game-based interventions on affective aspects included two subdomains: shifting and emotional control, as measured using the BRIEF. Compared with the control group, game-based interventions were not effective in improving shifting (MD = 0.42; 95% CI = [− 2.84, 3.68]) or emotional control (MD = 0.37; 95% CI = [− 3.16, 3.89]). Figure 6 presents the results of the meta-analysis on affective outcomes.

## Discussion

To the best of our knowledge, this is the first study to systematically integrate the strategies and effects of game-based digital interventions for children and adolescents with ADHD within the framework of ELT. While previous studies have examined the effectiveness of digital interventions, they often lacked a unified theoretical perspective, making it difficult to generalize findings. By applying ELT, our study provides a structured understanding of how these interventions influence cognitive, behavioral, and affective outcomes in children and adolescents with ADHD.

Given that cognitive function is central to ADHD treatment, most studies in this review developed games specifically designed to enhance cognitive abilities and utilized diverse tools to assess their outcomes. ADHD is characterized by deficits in executive function, including impaired impulse control, attention regulation, and working memory, which significantly impact daily functioning [38]. Addressing these deficits through targeted cognitive interventions is therefore a key therapeutic goal.

Game-based interventions have been recognized for their potential to improve cognitive function by engaging specific neural systems involved in attention, memory, and executive function [21, 39, 40]. Tools such as the Continuous Performance Test and Stroop Test were used to measure executive function and attentional control, while the Wechsler Intelligence Scale for Children, particularly its Working Memory Index (WMI), assessed working memory, which is closely linked to ADHD symptoms [41–43]. The results of our review indicate that game-based interventions yielded significant improvements in both forward and backward working memory tasks, as demonstrated through CBTT analyses, with statistically meaningful effect sizes. These findings support the theoretical premise that repetitive and adaptive game-based training can enhance core cognitive processes [39, 44].

However, when compared to pharmacological treatments, these effects appear to be more domain specific. A meta-analysis on methylphenidate (MPH) compared to placebo demonstrated significant improvements across multiple cognitive domains, including executive memory, non-executive memory, reaction time, and response inhibition [45]. Previous research has shown that stimulant medications generally produce broader and more substantial improvements in both ADHD symptoms and cognitive performance, particularly in executive function and impulse control. In contrast, the cognitive benefits of game-based interventions tend to be more targeted, with improvements primarily observed in working memory rather than across multiple domains. These findings suggest that while MPH broadly enhances cognitive control, game-based interventions may provide more specific enhancements in visuospatial working memory.

Despite the cognitive benefits of stimulant medications, they are often associated with side effects such as appetite suppression, growth retardation, weight loss, increased blood pressure, elevated heart rate, sleep disturbances, and tics [46]. Given these concerns, recent clinical guidelines emphasize that ADHD should be managed as a chronic condition rather than a disorder that can be cured solely through medication [46]. Consequently, non-pharmacological interventions are recommended as the first-line treatment, particularly in younger children, with a combination of medication and behavioral interventions being advised for optimal management when necessary [46]. Future research should therefore explore whether integrating digital interventions with pharmacological treatments can yield complementary benefits in ADHD care.

However, one of the major limitations observed in this review is the lack of standardized tools and methodologies across game-based intervention studies, which complicates direct comparisons of outcomes. Variability in study designs, intervention protocols, and cognitive assessment tools limits the ability to draw definitive conclusions regarding their efficacy. Future research should aim to develop a core set of outcome measures to ensure consistency and reproducibility, thereby improving the comparability and generalizability of findings in this field.

Behavioral responses are a key consideration in interventions for children with ADHD, with many approaches aiming to increase desired behaviors (e.g., compliance) and reduce undesirable ones (e.g., noncompliance) [47]. The effectiveness of game-based interventions on behavioral outcomes was mainly assessed using the BRIEF in this review. While six of the eight studies used the BRIEF, no significant differences were found across its subscales. As the most frequently used tool in this analysis, the BRIEF consists of nine subscales and is designed to assess how executive functions manifest through behavior in real-life settings. However, many studies focused on specific subscales of the BRIEF to evaluate particular domains, rather than using the full tool for a more integrated assessment [48, 49]. This approach may limit the ability to capture comprehensive behavioral effects. Another potential factor could be the complex interaction between cognition and behavior that the BRIEF attempts to measure. Behavioral manifestations of cognitive function are multifaceted and influenced by various contextual factors, making it difficult to directly assess intervention effects. Additionally, the relatively young age of the participants (6–12 years) may have influenced the results, as behavioral changes at this developmental stage can be subtle or variable over time. Therefore, longitudinal studies using objective evaluation methods are essential for understanding the behavioral changes and long-term effects of game-based interventions.

However, emotional outcomes were not fully addressed in this study owing to the limited number of studies focusing on the affective domain. Given the nature of ADHD, addressing emotional aspects through game-based interventions is critical. Children with ADHD often struggle with emotional regulation, which significantly affects their social interactions and quality of life [50, 51]. Owing to their engaging and adaptive nature, game-based interventions have the potential to positively impact emotional well-being by fostering self-efficacy, improving frustration tolerance, and promoting intrinsic motivation. Future research should explore how game-based interventions can be specifically designed to target emotional outcomes, ensuring a more comprehensive approach to managing ADHD symptoms.

Finally, this review identified several game elements, such as points, badges, rewards, customizable levels, and narrative-driven stories, used in game-based digital interventions for ADHD. Since motivation is a crucial factor in children with ADHD, providing an optimal level of challenge tailored to individual performance is particularly effective in maintaining engagement [52]. With advancements in technology, artificial intelligence now allows for real-time difficulty adjustments, as seen in platforms like EndeavorRx [53]. These dynamic adjustments ensure that games remain engaging and personalized, maximizing their therapeutic impact. Additionally, the lack of objectivity in task performance is often linked with cognitive impairments in ADHD. Therefore, consistently providing clear and direct feedback as players progress through game-based digital interventions is essential [54]. Clear, consistent feedback helps participants stay focused, enhances their understanding of their progress, and reinforces motivation.

This study has several strengths in evaluating the effectiveness of game-based digital interventions for children and adolescents with ADHD. By including only RCTs, it ensures high reliability and internal validity. Furthermore, interpreting the findings through Beard and Wilson’s ELT provides a solid theoretical framework for the analysis. By examining cognitive, behavioral, and affective outcomes, this study offers a comprehensive understanding of the potential efficacy of game-based interventions. However, this study has limitations. First, the inclusion of only eight studies resulted in a relatively small sample size, limiting the generalizability of the findings. Second, the diverse range of measurement tools used across studies presents challenges in synthesizing results and drawing consistent conclusions.

## Conclusion

Over the past decade, the use of game-based technologies in school-age children has gained attention as a non-pharmacological treatment for ADHD. However, the effectiveness of game-based digital interventions for children and adolescents remains unclear. To our knowledge, this is the first systematic review and meta-analysis of RCTs that integrates and evaluates the efficacy of game-based interventions grounded in ELT. By synthesizing evidence from eight RCTs, the findings suggest that game-based interventions can enhance visuospatial short-term memory and working memory—key components of the cognitive domain. Despite the limited number of RCTs, this study underscores the potential of game-based interventions as a safe, engaging, and non-pharmacological approach to improving ADHD symptoms, without the side effects associated with medication. While most research has focused on cognitive effects, future studies should aim to develop standardized assessment frameworks, utilize objective evaluation methods, and conduct longitudinal research to better understand the behavioral and long-term outcomes. These efforts will help design interventions that address cognitive deficits, as well as emotional and social challenges.

## Electronic supplementary material

Below is the link to the electronic supplementary material.

Supplementary Material 1

## Data Availability

No datasets were generated or analysed during the current study.
