# Supplementary material for: Effectiveness of game-based digital intervention for attention-deficit hyperactivity disorder in children and adolescents: a systematic review and meta-analysis using Beard and Wilson’s conceptualization of perception in experiential learning
Source: Eur Child Adolesc Psychiatry. 2025 Jun 16;34(12):3853–69. doi: 10.1007/s00787-025-02788-5 (PMC12743074; doi:10.1007/s00787-025-02788-5)
Supplement: Supplementary file 1 — Supplementary Material 1 [file 787_2025_2788_MOESM1_ESM.docx]

**Supplementary table 1** Search Strategy for Systematic Review on Game-Based Digital Interventions for ADHD in Children and Adolescents

| Keywords | MeSH or Emtree | Search terms |
| --- | --- | --- |
| Children and adolescents with ADHD (P) | attention deficit hyperactivity disorder (P_1) | Attention Deficit Disorder with Hyperactivity, Attention Deficit Disorder*, Hyperactivity Disorder Attention Deficit Hyperactivity Disorder*, ADHD, Attention Deficit-Hyperactivity Disorder*, ADDH, Deficit-Hyperactivity Disorder, Attention, Hyperkinetic Syndrome, Minimal Brain Dysfunction, Minimal Brain Disorder*, Minimal Brain Damage*, Chronic Brain Damage*, Chronic Encephalopathy, hyperactiv*, hyperkinesis* |
|  | Child, adolescent (P_2) | Child*, Adolescen*, Juvenile*, Pediatric*, Paediatric*, School age*, Schoolage*, Teen*, youth, school children |
| Game-based  digital  intervention (I) | Digital health (I_1) | Digital Health*, digital intervention, digital digital thera*, eHealth Therapy, Computer-Assisted, Computer-Assisted Therap*, Computer-Assisted Protocol-Directed Therap* |
|  | Therapy, Computer-Assisted (I_2) | Computer based treatment protocol, Active-Video, Virtual Reality exercis* |
|  | Mobile Applications,  Cell Phone,  Computers, Handheld (I_3) | Mobile Application*, Cell Phone*, “Computers, Handheld mobile phone*”, personal digital assistant, Cellular Phone*, Cellular Telephone*, Mobile Telephone*, Smart phone*, Tablet*, iphone*, ipad*, android*, Personal Digital Assistant*, Mobile Device*, tablet app*, Mobile App*, Portable Electronic App*, Portable Software App*, Smartphone App*, m-learn*, mlearn*, m‐health, mhealth, mobile health |
|  | Game (I_4) | Game, serious game*, gaming play, Computer-Assisted Video Game*, Computer game based*, Game based exercis*, Exergam*, Play Therap*, Video game*, Computer Game*, Electronic game*, Active Gam* |

**Supplementary table 2** PubMed search strategy

| **No** | **Query** | **Results** |
| --- | --- | --- |
| 1 | "Attention Deficit Disorder with Hyperactivity"[Title/Abstract] OR "attention deficit disorder*"[Title/Abstract] OR "Hyperactivity Disorder Attention Deficit"[Title/Abstract] OR "hyperactivity disorder*"[Title/Abstract] OR "ADHD"[Title/Abstract] OR "attention deficit hyperactivity disorder*"[Title/Abstract] OR "ADDH"[Title/Abstract] OR "deficit hyperactivity disorder attention"[Title/Abstract] OR "Hyperkinetic Syndrome"[Title/Abstract] OR "Minimal Brain Dysfunction"[Title/Abstract] OR "minimal brain disorder*"[Title/Abstract] OR "minimal brain damage*"[Title/Abstract] OR "chronic brain damage*"[Title/Abstract] OR "Chronic Encephalopathy"[Title/Abstract] OR "hyperactiv*"[Title/Abstract] OR "hyperkinesis*"[Title/Abstract] | 89,635 |
| 2 | "child*"[Title/Abstract] OR "adolescen*"[Title/Abstract] OR "juvenile*"[Title/Abstract] OR "pediatric*"[Title/Abstract] OR "paediatric*"[Title/Abstract] OR "school age*"[Title/Abstract] OR "schoolage*"[Title/Abstract] OR "teen*"[Title/Abstract] OR "youth"[Title/Abstract] OR "school children"[Title/Abstract] | 2,331,147 |
| 3 | #1 AND #2 | 37,214 |
| 4 | "digital health*"[Title/Abstract] OR "digital intervention"[Title/Abstract] OR "digital"[Title/Abstract] OR "digital thera*"[Title/Abstract] OR "eHealth"[Title/Abstract] OR "therapy computer assisted"[Title/Abstract] OR "computer assisted therap*"[Title/Abstract] OR "computer assisted protocol directed therap*"[Title/Abstract] | 218,534 |
| 5 | ("Computer based"[Title/Abstract] AND "treatment protocol"[Title/Abstract]) OR "Active-Video"[Title/Abstract] OR "virtual reality exercis*"[Title/Abstract] | 533 |
| 6 | "mobile application*"[Title/Abstract] OR "cell phone*"[Title/Abstract] OR "computers handheld"[Title/Abstract] OR "mobile phone*"[Title/Abstract] OR "personal digital assistant"[Title/Abstract] OR "cellular phone*"[Title/Abstract] OR "cellular telephone*"[Title/Abstract] OR "mobile telephone*"[Title/Abstract] OR "smart phone*"[Title/Abstract] OR "tablet*"[Title/Abstract] OR "iphone*"[Title/Abstract] OR "ipad*"[Title/Abstract] OR "android*"[Title/Abstract] OR "personal digital assistant*"[Title/Abstract] OR "mobile device*"[Title/Abstract] OR "tablet app*"[Title/Abstract] OR "mobile app*"[Title/Abstract] OR "portable electronic app*"[Title/Abstract] OR "portable software app*"[Title/Abstract] OR "smartphone app*"[Title/Abstract] OR "m learn*"[Title/Abstract] OR "mlearn*"[Title/Abstract] OR "m-health"[Title/Abstract] OR "mhealth"[Title/Abstract] OR "mobile health"[Title/Abstract] | 124,145 |
| 7 | "game"[Title/Abstract] OR "serious game*"[Title/Abstract] OR "computer game based*"[Title/Abstract] OR "game based exercis*"[Title/Abstract] OR "exergam*"[Title/Abstract] OR "play therap*"[Title/Abstract] OR "video game*"[Title/Abstract] OR "computer game*"[Title/Abstract] OR "electronic game*"[Title/Abstract] OR "active gam*"[Title/Abstract] | 329,810 |
| 8 | #4 or #5 or #6 | 43,752 |
| 9 | #7 and #8 | 2,931 |
| 10 | #3 and #9 | 49 |

**Supplementary table 3** Gaming elements and strategies in the reviewed studies

| No | Author  (year) | Gaming elements and Strategies |  |
| --- | --- | --- | --- |
| 1 | Bikic  (2018) | Reward (points), Level of difficulty (assess to adaptive ability),  Virtual Goods, Progress |  |
| 2 | Dovis  (2015) | Reward (badges), Level of difficulty (assess to adaptive ability),  Avatars, Story, Progress |  |
| 3 | Dovis  (2019) | Reward (badges), Level of difficulty (assess to adaptive ability),  Avatars, Story, Progress |  |
| 4 | Kim  (2016) | Reward (badges), Mission (quests or challenges), Virtual Goods,  Social interaction, Leaderboards, Story, Progress |  |
| 5 | Kim  (2022) | AI-based program (not sure which gaming elements were embedded in the intervention) |  |
| 6 | Kollins  (2020) | Reward,  Level of difficulty tailored to the participant's abilities (in real time) |  |
| 7 | Medina  (2021) | Level of difficulty tailored to the participant's abilities (AI) |  |
| 8 | Moradi  (2024) | Leaderboards |  |
